# Chromatin remodeling in tissue stem cell fate determination

**DOI:** 10.1186/s13619-024-00203-z

**Published:** 2024-09-30

**Authors:** Xinyang Li, Gaoxiang Zhu, Bing Zhao

**Affiliations:** 1 State Key Laboratory of Genetic Engineering, School of Life Sciences, Fudan University, Shanghai, 200438 China; 2 School of Basic Medical Sciences, Jiangxi Medical College, The First Affiliated Hospital of Nanchang University, Nanchang University, Nanchang, 330031 China; 3 Z Lab, bioGenous BIOTECH, Shanghai, 200438 China

**Keywords:** Tissue stem cells, Chromatin remodeling, Cell fate determination

## Abstract

Tissue stem cells (TSCs), which reside in specialized tissues, constitute the major cell sources for tissue homeostasis and regeneration, and the contribution of transcriptional or epigenetic regulation of distinct biological processes in TSCs has been discussed in the past few decades. Meanwhile, ATP-dependent chromatin remodelers use the energy from ATP hydrolysis to remodel nucleosomes, thereby affecting chromatin dynamics and the regulation of gene expression programs in each cell type. However, the role of chromatin remodelers in tissue stem cell fate determination is less well understood. In this review, we systematically discuss recent advances in epigenetic control by chromatin remodelers of hematopoietic stem cells, intestinal epithelial stem cells, neural stem cells, and skin stem cells in their fate determination and highlight the importance of their essential role in tissue homeostasis, development, and regeneration. Moreover, the exploration of the molecular and cellular mechanisms of TSCs is crucial for advancing our understanding of tissue maintenance and for the discovery of novel therapeutic targets.

## Background

Stem cells are a class of cells with the capacity for self-renewal and multi-lineage differentiation. Based on the source of origin, stem cells can be classified as tissue stem cells (TSCs), embryonic stem cells, induced pluripotent stem cells, and other stem cells (Essawy et al. 2020; Shah et al. 2021). TSCs are a type of quiescent resident stem cells that remain in an undifferentiated state in most organs or tissues, playing an important role in maintaining tissue homeostasis, cell proliferation, differentiation, and aging. Their regenerative properties have garnered significant attention over the past seven decades. As biological technology advances (such as label-retention assays (Cotsarelis et al. 1990), lineage tracing labeling in vivo (Barker et al. 2007), function examination for proliferation and differentiation ability in vitro (Xin et al. 2007), evaluation of reconstruction of tissues in vivo (Biasco et al. 2016), different TSCs are being identified and characterized, including hematopoietic stem cells (HSCs) (Thomas et al. 1957), muscle satellite cells (MuSCs) (Mauro 1961), neural stem cells (NSCs) (Paton and Nottebohm 1984), hair follicle stem cells (HFSCs) (Cotsarelis et al. 1990), mesenchymal stem cells (MSCs) (Colter et al. 2001), intestinal epithelial stem cells (IESCs) (Barker et al. 2007), etc. Besides, new types of TSCs have also been continually discovered in recent years, such as Procr^+^ progenitors (Wang et al. 2020), Dist-Luminal-C luminal progenitor cells (Guo et al. 2020), zone 2 hepatocytes (He et al. 2021; Wei et al. 2021), Prrx1-expressing cells (Liu et al. 2022), alveolar stem cells (Liu et al. 2024), etc. This statement implies that TSCs have the capacity to self-renew and differentiate into distinct cell types, rendering them a desirable resource for regenerative therapy. The balance between self-renewal and differentiation, which contributes to the basic functions of TSCs, is regulated precisely (Liu et al. 2010; Sheaffer et al. 2014; Ezhkova et al. 2009); however, disruption of it can lead to the failure of maintaining tissue homeostasis and restoration during regeneration, which may ultimately lead to a variety of disorders and diseases, such as cancer, in which epigenetic mechanisms (e.g., chromatin remodeling) are extremely important by proper control of gene expression at critical loci in specific tissue stem cell type. For example, existing studies have shown that in vivo deficiency of the Tets family (important epigenetic factors that regulate DNA methylation) alters the hematopoietic stem cell pool and the subsequent differentiation program and ultimately develops into myeloid malignancies (Cimmino et al. 2015; Li et al. 2011), which indicates that epigenetic factors in TSCs act as tumor suppressor genes to prevent the occurrence of tumors or disease.

Here we review the multifaceted roles of chromatin remodelers in cell fate determination on HSCs, IESCs, NSCs, skin stem cells, and MSCs based on a brief introduction to the basic function of TSCs and four subfamilies of chromatin-remodeling enzymes, and discuss the implication of epigenetic regulation during cell fate determination of TSCs.

## Chromatin remodeling by chromatin remodelers

DNA and histone jointly constitute the fundamental structure of chromatin, namely the nucleosome, with the nucleosome core being composed of 147 bp of DNA wrapping around the histone octamer (Luger et al. 1997). The advancement in the study of nucleosomes and chromatin has offered detailed insights into the landscape of chromatin organization and dynamics (Lai and Pugh 2017). The regulation of chromatin dynamics could take place at distinct epigenetic layers, such as DNA methylation (5meC), post-translational histone modification, chromatin remodeling, specific histone variants, non-coding RNA, etc. As one of the major mechanisms for regulating chromatin dynamics and 3D genome structure, chromatin remodelers, these specialized ATP-dependent enzymes, play crucial roles in chromatin remodeling-mediated transcription regulation, which leads to significant implications in development, tissue homeostasis, and regeneration, as well as diseases.

### ATP-dependent chromatin remodelers

Multicellular organisms develop from the zygote into tissues and organs with specialized cells to perform specific functions, which are regulated by the gene-expression program facilitated by tissue-specific regulators. The traditional view has confirmed the great potential of transcription factors as master regulators in the determination of cell fate, especially in the field of cell reprogramming (Takahashi and Yamanaka 2006). Furthermore, transcription factors interact with other transcription regulators to remodel chromatin, and thus to achieve the precise temporal and spatial control of gene expression programs within an organism. Chromatin remodeling mediated by chromatin remodelers plays critical roles in a variety of cellular events in the context of chromatin, including transcriptional regulation, DNA replication, and repair (Morrison and Shen 2009), all of which are essential for mammalian development and cellular differentiation. The breakthroughs in biotechnology have made it possible for researchers to investigate the crucial role that chromatin remodeling plays in directing TSCs’ choices for cell fate during a variety of biological processes from a thorough and original point of view (Buenrostro et al. 2018; Tsompana and Buck 2014). As one of the epigenetic modifications, ATP-dependent chromatin remodelers can cooperate with other types of modifications, such as DNA methylation and histone acetyltransferases, to regulate gene transcription (Gibbons et al. 2000; Sanz et al. 2016), and chromatin remodelers use distinct mechanisms to regulate the selective transcription processes and signaling pathways, which further determine the cell fate. However, the function of chromatin remodeling-regulated tissue stem cell fate determination in tissue homeostasis and tissue repair following damage is less known. Furthermore, it is rapidly becoming clearer how chromatin remodelers collaborate with additional components to decide the fate of TSCs in a multilayered and intricate way.

Evolutionarily conserved from yeast to mammals, chromatin remodelers are categorized into four subfamilies (Ho and Crabtree 2010), namely INO80 (inositol requiring 80), ISWI (imitation switch), CHD (chromodomain helicase DNA-binding), and SWI/SNF (switch/sucrose non-fermentable), which assume pivotal roles in orchestrating chromatin dynamics during ontogeny development, tissue response to external stimuli, and disease progression. All chromatin remodelers possess an ATPase domain that serves as a motor for translocating DNA and diverse sets of other subunits as accessory proteins to assist the ATPase in fulfilling its catalytic activity. The specific function of chromatin remodelers is to utilize energy from ATP hydrolysis to drive a DNA translocase, which leads to either the sliding or ejecting of nucleosomes from precise locations in the genome (Clapier et al. 2017). Given that the major effects brought by chromatin remodelers are to modify the contacts between DNA and histones, different forms of these energy-dependent chromatin remodelers play an essential role in regulating transcription activation or repression. It is notable that chromatin remodelers exert non-redundant functions and are stringently regulated at specific developmental stages during organogenesis and postnatal development (Hota and Bruneau 2016). Specifically, tissues in adults seem to require a more elaborate regulatory program, which largely depends on the function of chromatin remodelers of diverse combinatorial assemblies that are different from the complexes in the embryonic stage in a tissue-dependent pattern. For instance, Chd7 plays a more significant role in the maintenance of neural stem cells during adult neurogenesis (Jones et al. 2015), based on the fact that adult neurogenesis occurs in specialized niches in the brain, such as the subgranular zone (SGZ) of the hippocampus and the subventricular zone (SVZ), and the mechanisms that govern adult neurogenesis are distinct from those in embryonic development (Feng et al. 2013; Micucci et al. 2014). Ablation of Brg1 in the surface ectoderm at E12.5 and epidermal keratinocytes does not affect their proliferation, and keratinocytes undergo normal stratification; however, their late terminal differentiation is impaired, which finally leads to postnatal death (Indra et al. 2005). Also, Brg1 has distinct roles in embryonic cardiomyocytes compared with adult cardiomyocytes to regulate gene expression shifts (Hang et al. 2010). Different forms of combinatorial assembly of each complex are required to guide the dynamic chromatin organization but will not be discussed in detail. Subsequently, we will focus on the fundamental functions of the four subfamilies and illustrate their predominant role in regulating chromatin accessibility.

### Action model of chromatin remodelers

Although each subfamily exerts differential effects on chromatin structure, these enzymes basically regulate nucleosome “behaviors” by influencing nucleosome composition or positioning, which ultimately leads to the alteration of chromatin state. Specific domains in each complex of four subfamilies perform distinct functions. For instance, the ATPase domain for each subfamily serves as the catalytic subunit to achieve DNA translocation by disrupting histone-DNA contacts (Clapier and Cairns 2009). The major function in chromatin regulation among four subfamilies is diverse, with an emphasis on the specific action mode for each subfamily. Briefly, the ISWI and CHD subfamily remodelers are accountable for nucleosome assembly during DNA replication subsequent to the deposition of histone complexes by histone chaperones, and the stipulation to establish sliding and spacing activities is also requisite to exert their effects in transcription (Ocampo et al. 2016). The SWI/SNF subfamily is implicated in ejecting nucleosome components or sliding nucleosomes along DNA to achieve irregular spacing, which results in accessible chromatin landscapes. For INO80 remodelers, besides their role in regulating nucleosome spacing (Udugama et al. 2011), the unique function of this subfamily to substitute canonical histones with histone variants during transcription and DNA repair could greatly alter chromatin state by influencing factor recruitment, exclusion, and activity. For example, the SRCAP chromatin remodeling complex executes ATP-dependent activity to deposit the H2A.Z-H2B dimer into nucleosomes with the eviction of H2A-H2B for the promotion of gene expression (Liang et al. 2016; Luk et al. 2010; Mizuguchi et al. 2004). Even though these chromatin remodelers execute distinct roles in altering chromatin structure, it is reported that all these remodelers share the same mechanisms to drive DNA translocation, as demonstrated by a model proposed by Velankar et al., indicating an ‘Inchworming’ mechanism mediated by the ATPase domains to drive DNA translocation, whereby the shared lobe1 and lobe2 for each ATPase subunit can directly bind to the DNA and the subsequent unidirectional movement results in 1–2 bp in each ATP hydrolysis cycle (Velankar et al. 1999). The specific mode of these chromatin-remodeling complexes prompts researchers to further report the resolved structure of them. Taking the extensively studied subfamily SWI/SNF as an example, biologists determined the higher-level resolution of this family based on the basic module using cryo-electron microscopy (cryo-EM) (He et al. 2020; Wang et al. 2022; Yuan et al. 2022), which informs the mechanisms of how subunit mutation could ultimately lead to human diseases (Mashtalir et al. 2020; Valencia et al. 2019; Farnung et al. 2020).

### Chromatin accessibility by chromatin remodelers

**Fig. 1:**
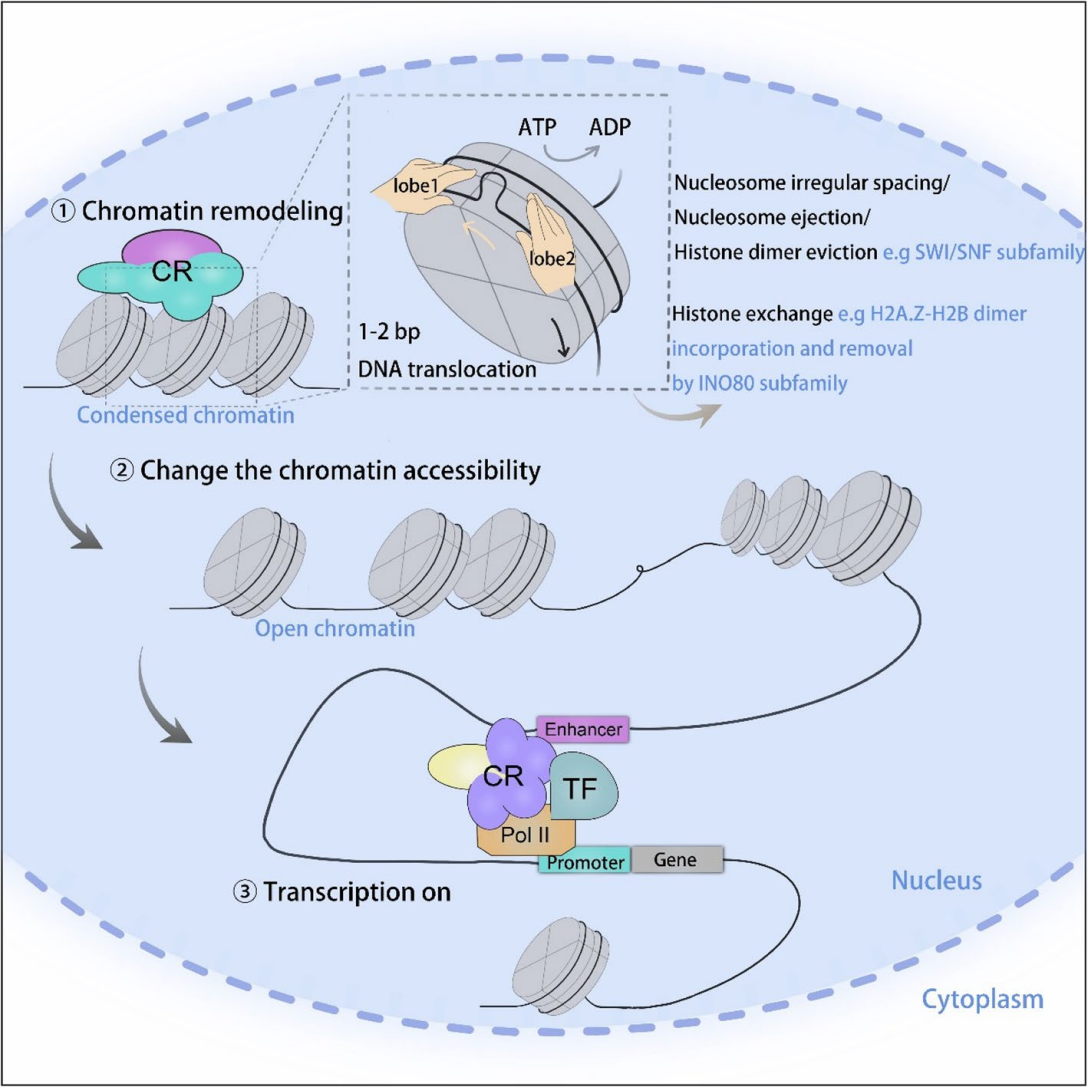
Chromatin remodelers are major contributors to epigenetically regulate chromatin accessibility and transcription. Nucleosomes are organized into highly condensed chromatin which makes it inaccessible to regulators controlling gene expression. Once chromatin remodelers are recruited to the specific genome loci, lobe1 and lobe2 sequentially bind to the DNA to initiate DNA translocation, causing the DNA “wave” and the unidirectional movement of DNA around the octamer surface of 1–2 bp every cycle for ATP binding and hydrolysis, followed by the cooperation with other subunits, to the differential results including nucleosome spacing, ejection, histone dimer eviction, and histone exchange, which finally results in the loosening of the chromatin structure. The open chromatin configuration acts as a pre-requisite for gene transcription to allow access to transcriptional regulators. CR: chromatin remodeler; TF: transcription factor

The regulation of chromatin dynamics holds great significance for Pol II-mediated transcription, within which chromatin remodelers play essential roles in both the initiation and elongation processes (Li et al. 2007). The transcription process is largely dependent on chromatin accessibility, which is defined by the accessible regions in the genome that can perform regulatory functions for the binding of transcription factors and diverse protein factors. Chromatin accessibility is mainly regulated by histone-modifying enzymes and ATP-dependent enzymes. Histones are frequently modified by distinct enzymes to determine chromatin structure, thereby either preventing or enabling the access of specific proteins to chromatin (Kouzarides 2007). As aforementioned, the nucleosomal DNA translocation mediated by chromatin remodelers can also alter chromatin structure by disrupting the contacts between DNA and histones, thereby providing chromatin accessibility for gene transcription. Regarding transcription regulation, chromatin remodelers in the INO80 family regulate the process of histone variant exchange, which is crucial for controlling gene expression and chromatin accessibility (Watanabe and Peterson 2010). Specifically, the p400 and SRCAP remodelers are responsible for the incorporation of the histone variant H2A.Z into nucleosomes, thereby altering the local chromatin structure and facilitating transcription initiation and elongation. On the other hand, the INO80 subfamily of remodelers has been extensively investigated for its role in ejecting H2A.Z from nucleosomes. This mechanism is critical for maintaining the dynamic balance of H2A.Z distribution within the genome, which in turn affects the accessibility of DNA to transcriptional machinery. The removal of H2A.Z by INO80 remodelers can either repress transcription by stabilizing nucleosome occupancy or activate transcription by promoting the assembly of pre-initiation complexes at gene promoters (Martire and Banaszynski 2020). Notably, the SWI/SNF subfamily is crucial in establishing chromatin accessibility by sliding or evicting the nucleosome components to expose particular DNA sequences that allow the binding of the transcriptional machinery at promoters and enhancers (Boeger et al. 2004; Hargreaves and Crabtree 2011). Additionally, in transcription-associated processes, the interaction between histone-modifying enzymes and ATP-dependent enzymes involves the incorporation of different histone variants (Venkatesh and Workman 2015). In a word, the landscape of chromatin structure under epigenetic control requires different sets of regulators to function synergistically, to jointly ensure regular dynamics of chromatin structure and organization, manifested as a change in chromatin accessibility (Fig. 1).

Given the fundamental significance of chromatin accessibility for RNA polymerase II-mediated transcription, DNA replication, and repair, it has been reported that chromatin accessibility is regulated at multiple levels within the eukaryotic genome. These levels encompass nucleosome occupancy, density, and turnover, along with the involvement of linker histones and other architectural proteins, and the three-dimensional (3D) organization of the genome (Klemm et al. 2019). During specific stages within these biological processes, the regulation mediated by chromatin remodelers makes a substantial contribution. It is particularly interesting to understand precisely how these remodelers are implicated in regulating these processes. For example, the NuRD complex, which is one of the ISWI complexes, participates in the displacement of histone H1, which serves as a repressor of transcription. This displacement is a prerequisite for the activation of hormone-responsive genes. In this context, the NuRD complex is initially recruited by the activated progesterone receptor (PR) and subsequently facilitates the PR-mediated recruitment of Cdk2/CyclinA for the displacement of histone H1 (Vicent et al. 2011). The ISWI and CHD subfamilies perform indispensable functions in nucleosome assembly and spacing, enabling the correct organization of chromatin during DNA replication (Yadav and Whitehouse 2016). Specifically, ISWI complexes assist in evenly spacing nucleosomes along the DNA strand, which is crucial for the proper advancement of the replication fork. Meanwhile, members of the CHD family are involved in the assembly and disassembly of nucleosomes, contributing to the maintenance of chromatin structure and accessibility. Furthermore, among the diverse pathways of DNA repair, different chromatin remodeler complexes exert specific molecular activities to regulate different steps of the DNA damage response (DDR) (Lans et al. 2012). For instance, SWI/SNF complexes are known to facilitate the access of repair enzymes to sites of DNA damage by altering nucleosome positioning. Similarly, INO80 and SWR1 complexes play roles in the exchange of histone variants and the repair of double-strand breaks.

## Tissue stem cells

**Fig. 2:**
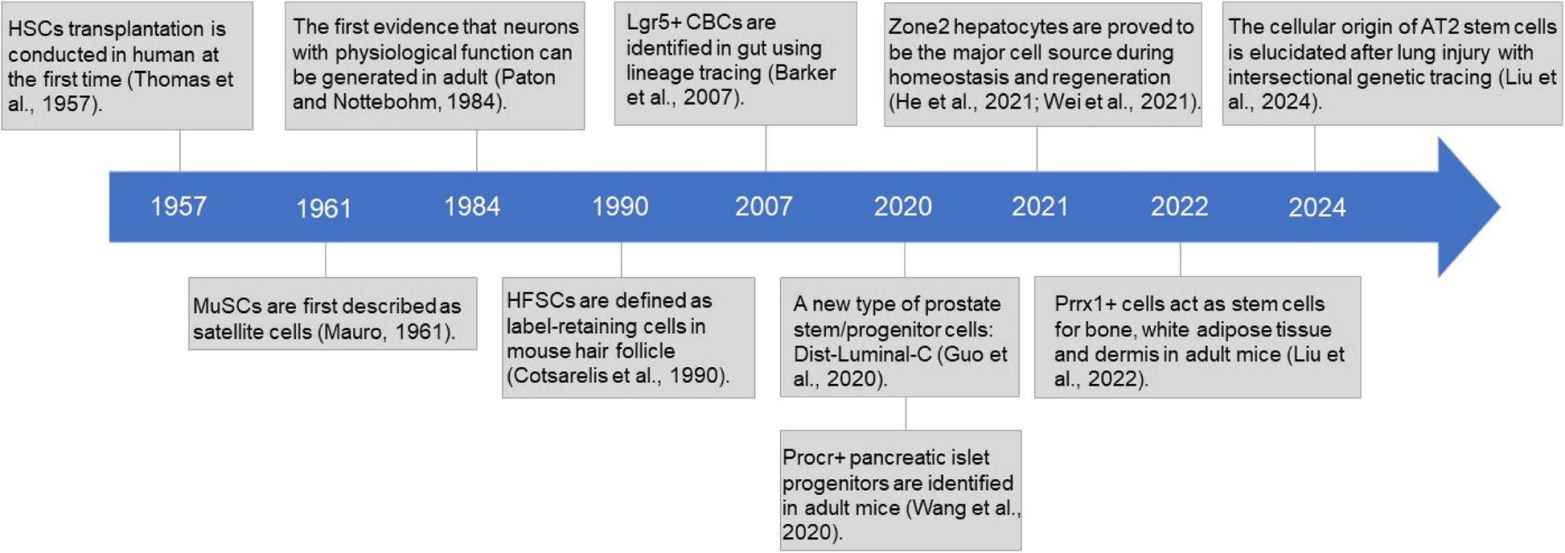
History of progress in the field of tissue stem cells

In the context of stem cell research, pluripotent stem cells, such as embryonic stem cells (ESCs) and induced pluripotent stem cells (iPSCs), can be stably passaged and maintained in an undifferentiated state, with the inherent potential to differentiate into any type of somatic cell, which makes them invaluable tools for regenerative medicine, drug discovery, and disease modeling. However, this comes with certain challenges and limitations. Because they are associated with the risks of tumorigenicity, due to their unlimited proliferative capacity lack of differentiation control, and a dearth of tissue specificity, the therapeutic use of them is limited. In contradistinction to these cells, TSCs, also known as adult stem cells, begin their role in the 1950s (as shown in Fig. 2), and assume a critical role in tissue homeostasis and regeneration (Fu et al. 2021). These cells are found within various tissues and organs and are responsible for maintaining the integrity and functionality of these tissues throughout an organism’s lifetime. They are usually in specific microenvironments and are precisely regulated by various signaling molecules and intercellular interactions to maintain their stem cell characteristics and appropriate differentiation capabilities. The characteristics of TSCs enable them to play a key role in the growth, repair, and maintenance of tissues. Unlike pluripotent stem cells, which can differentiate into any cell type in the body, TSCs have a more limited differentiation potential, typically restricted to the cell types present in their tissue of origin. This tissue-specific differentiation capacity allows them to efficiently replace damaged or lost cells while minimizing the risk of forming tumors, which renders the use of TSCs in translational research relatively safer (Wang et al. 2023).

### Basic behavior and function of tissue stem cells

**Fig. 3:**
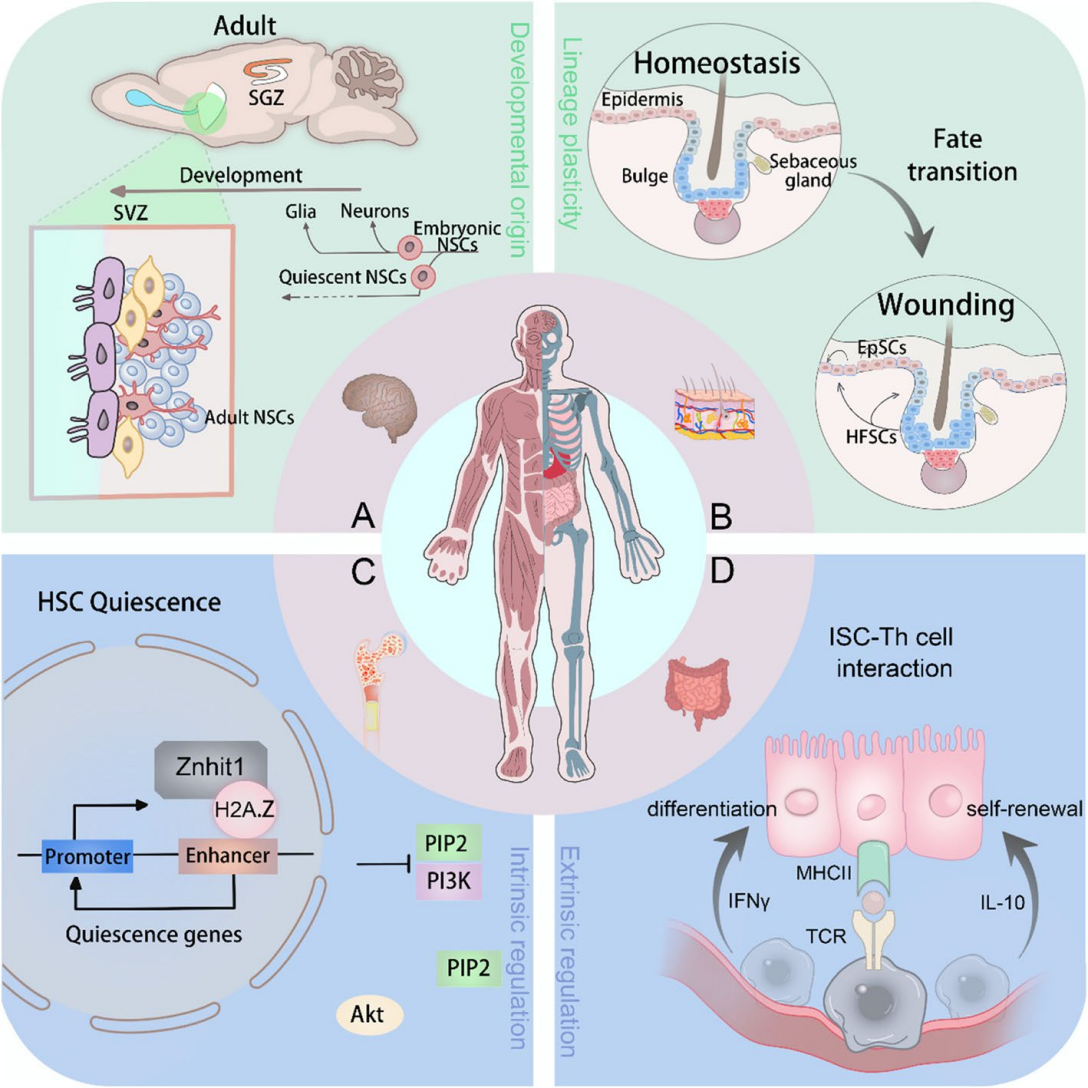
Characterization of tissue stem cells. **A** Identifying where TSCs come from is critical to understanding their properties. Adult NSCs in mammals are quiescent and have two distinct origins. In contrast to SGZ NSCs, SVZ NSCs generate from the slowly dividing neural progenitors in SVZ between E13.5-E15.5. These stem cells are reactivated in postnatal life for the production of olfactory bulb interneurons. **B** Lineage plasticity in TSCs leads to a fate transition from HFSCs to EpSCs upon injury, in which HFSCs are recruited to the epidermis to repair the tissue. **C** Tissue stem cell fate determination can be regulated by intrinsic factors. HSC quiescence is regulated by the chromatin remodeler Znhit1. **D** Tissue stem cell fate determination can be regulated by extrinsic factors. Lgr5^+^ CBCs interact with T helper cells to regulate self-renewal and differentiation into distinct lineages. EpSCs: epidermal stem cells; MHCII: major histocompatibility complex class II; TCR: T cell receptor

Understanding the function of specific TSCs and the progeny they can produce within an adult context is predominantly reliant on an understanding of their developmental origin, which underpins the appropriate establishment of TSCs. For example, the generation of adult NSCs from embryonic progenitor cells seemingly follows dissimilar models; SVZ NSCs emanate from a quiescent population that is set aside in the LV during E13.5 to E15.5, while SGZ NSCs originate from continuously evolving progenitors (Cai and Yang 2019) (Fig. 3A). Although TSCs are a rare population of stem cells in the body, they have garnered significant attention in the past few decades due to their tremendous potential in regenerative medicine. These somatic stem cells exhibit shared fundamental behaviors, such as self-renewal, differentiation, quiescence, etc., which are regulated by both intrinsic cellular mechanisms and signals from specialized microenvironments. They serve as a group of resident cells with the ability to maintain tissue homeostasis and to restore and regenerate tissue responding to damage. A precisely regulated balance between the proliferation and differentiation of TSCs is requisite for normal development. Asymmetric cell division, being one of the fundamental properties of TSCs, is influenced by both extrinsic and intrinsic effectors and confers TSCs with the ability to balance between self-renewal and differentiation (Tajbakhsh et al. 2009). The immortal strand hypothesis has been proposed to suggest that the stem daughter cell always contains the older DNA to prevent possible errors during replication as asymmetric cell divisions occurs (Rando 2007), which is supported by long-term tracing of label-retaining cells in multiple systems (Karpowicz et al. 2005; Shinin et al. 2006; Smith 2005). However, the capacity for self-renewal in stem cells is not solely dependent on this traditional model. It seems to be asymmetric at the cell population level, but not at the level of single-cell division, because each stem cell has the same probability of self-renewal or differentiation. TSCs compete with each other to either survive or become extinct, which largely depends on the niche recording to a neutral drift model (Lopez-Garcia et al. 2010). It is reported that TSCs are often in a relatively quiescent state, which enables the detection of their existence using the long-term tracing method (Grompe 2012). The inactive state for stem cells to maintain quiescence controlled by unique features such as low metabolic activity and post-transcriptional regulation depicts significant potential in the regulation of stem cell quiescence (de Morree and Rando 2023). Lgr5^+^ crypt base columnar cells (CBCs) reside in intestinal crypts and remain long-term activity, akin to HSCs, yet can constantly give rise to lineage-restricted progeny cells to maintain intestinal homeostasis (Barker et al. 2007), while stem cells at the + 4 position have quiescent features and can be mobilized to restore the pool of Lgr5^+^ CBCs following irradiation-induced injury (Takeda et al. 2011). Besides, MuSCs, liver stem cells, and HFSCs with quiescent features can also be rapidly activated at the time of injury (Dumont et al. 2015; Ito et al. 2005; Tarlow et al. 2014). Despite the different states of quiescent and active stem cells under specific circumstances, the behaviors of these stem cells largely depend on the located microenvironment and the existence of both the active and quiescent populations to cooperate with each other, playing functional roles in a given tissue (Li and Clevers 2010). Remarkably, the lineage plasticity of TSCs to switch fates is well established in many tissues. For example, HFSCs that reside in the bulge area are recruited to the epidermis and reprogrammed to an interfollicular epidermis fate after injury (Ito et al. 2005) (Fig. 3B). Taken together, the fundamental function of these stem cells under different circumstances is to replace the lost cells in the tissue where they reside to maintain tissue integrity under homeostasis and regeneration upon injury (Post and Clevers 2019).

### Regulation of tissue stem cells

Due to the rapid advancement of new tools and technologies, nearly all types of TSCs in vivo have been identified by people through the utilization of diverse animal models to illustrate the mechanisms regarding their fundamental behaviors such as proliferation and differentiation. Our current understanding of the regulation of TSCs originates from a multitude of studies involving mechanisms at various levels in the control of tissue stem cell functions, cell fate determination, quiescence maintenance, and tissue regeneration, among others.

It is known that TSCs, which reside in specialized environments within tissues, receive local signals for modulating their behaviors, among which many growth factors serve as important signaling molecules, cooperating with intrinsic regulators. From the perspective of the intrinsic regulation of tissue stem cell fate, discoveries have illuminated the remarkable significance of metabolic networks in the governing of the chromatin landscape, giving rise to profound effects on the function of TSCs to differentiate (Intlekofer and Finley 2019). The bidirectional link between the metabolic pathways accountable for the introduction of substrates and co-factors for chromatin-modifying enzymes and the gene expression mediated by chromatin-modifying enzymes needs to be well established to avoid dysregulation (Arrowsmith et al. 2012; Kaelin and McKnight 2013). Stem cell niches are organized by specialized cells to regulate the maintenance of TSCs and their functions. For example, Th cells interact with the MHCII system in Lgr5^+^ ISCs to modulate the maintenance of the stem cell pool and differentiation during both homeostasis and infection (Biton et al. 2018) (Fig. 3D). Of note, the cooperation of intrinsic and extrinsic factors in the regulation of cell fate determination in TSCs often leads to different outcomes within a diverse context of adult tissues. As the only liquid organ in our body, the hematopoietic system requires HSCs to remain in a quiescent state to fulfill their function. The maintenance of a quiescent state could be regulated by intrinsic molecules (such as chromatin remodelers) and environmental signals, both of which are necessary to preserve genomic integrity and to maintain a poised state for activation (Cheung and Rando 2013). Significantly, both proliferation and dominant long-term quiescence in HSCs are under the precise control of intrinsic factors and external signals (Biermann and Reya 2022) (Fig. 3C). Moreover, the balance between quiescence and activity in NSCs to affect the long-term maintenance of the stem cell pool and adult neurogenesis is also characterized and regulated by intrinsic and extrinsic mechanisms (Urbán et al. 2019). TSCs like ISCs and HFSCs exposed to an external environment are more prone to damage. The maintenance of homeostasis and the ability to regenerate after injury in these cells are thus more stringently regulated by intrinsic and niche-derived regulatory signals.

## Chromatin remodeling in tissue stem cell fate determination

Cell fate determination is a fundamental issue in biology. Epigenetic control of cell fate determination has been investigated in extensive tissues under diverse settings of regulation, among which chromatin remodeler-mediated gene expression significantly underpins cell fate determination. Hereinafter, the role of chromatin remodelers in the hematopoietic system, the gut, the neural system, and the skin was summarized (Tables 1 and 2), with a focus on how chromatin remodelers carry out their functions in TSCs and eventually lead to the reconfiguration of tissue behavior, which offers us deeper insights into the role of chromatin remodeling in development, disease disorders, and tissue regeneration. Moreover, it will also help us further disclose how these fundamental biological processes mediated by TSCs are subjected to chromatin remodeler-controlled epigenetic regulation.

**Table 1:** Roles of SWI/SNF subfamily in tissue stem cells

Subunit	Cre driver lines	Phenotype in development, regeneration and diseases
BRG1	*Homozygous mutants VillinCreERT2; Lgr5-GFP-CreERT2* *Villin-Cre* *K14-CreERT2* *Nfatc1-Cre* *Nestin-Cre* *Emx1-Cre; Bhlhb5-Cre* *Nestin-CreERT2; R26R-YFP* *Nestin-Cre* *Olig1-Cre* *Glast-CreERT2*	Brg1 is required for definitive hematopoiesis (Tu et al. 2020), self-renewal capacity and differentiation for ISCs (Holik et al. 2013; Takada et al. 2016), differentiation of epithelial progenitor cells during development (Mardaryev et al. 2014), and for HFSCs in tissue regeneration and repair (Xiong et al. 2013). Brg1 deletion leads to failure of the maintenance and differentiation of NSCs (Jin et al. 2022; Matsumoto et al. 2006; Petrik et al. 2015), and the specification and differentiation of OPCs during embryonic development (Matsumoto et al. 2016; Yu et al. 2013), besides, the maintenance of the neurogenic lineage in the adult brain (Ninkovic et al. 2013).
BRM	*Homozygous mutants*	Brm regulates self-renewal of HSPCs (Naidu et al. 2022).
BAF53a	*Mx1-Cre* *K14-Cre*	BAF53a is essential for adult hemopoiesis (Krasteva et al. 2012) and the maintenance and differentiation of epidermal progenitors (Bao et al. 2013).
BAF45a	*Mx1-Cre*	BAF45a is dose-dependent required for HSCs maintenance and myeloid lineage commitment (Krasteva et al. 2017).
BAF180	*Rosa26-CreERT2*	BAF180 deletion results in decreased HSCs reconstitution ability partially by p21 (Lee et al. 2016).
BAF200	*Tie2-Cre; Vav-iCre; Mx1-Cre*	BAF200 is essential for normal hematopoiesis (Liu et al. 2018).
	*Vav1-Cre; Mx1-Cre*	BAF200 is almost dispensable for normal hematopoiesis but is required for HSCs differentiation upon BM transplantation (Bluemn et al. 2021).
BAF250a	*Mx1-Cre* *Villin-Cre; Lgr5CreERT2* *Emx1-Cre* *Cx3cr1-Cre*	BAF250a is required for the maintenance of HSCs quiescence (Han et al. 2019) and self-renewal capacity of ISCs (Hiramatsu et al. 2019). BAF250a regulates the self-renewal and differentiation of NSCs by cell-autonomous (Liu et al. 2021) and cell-non-autonomous (Su et al. 2024) mechanisms.
BAF250b	*Mx1-Cre*	BAF250b is required for the myeloid commitment in BM transplantation assay (Madan et al. 2023).
BRD9	*Mx1-Cre*	Brd9 is required for both normal and malignant hematopoiesis (Xiao et al. 2023).
BCL11B	*Lgr5-CreERT2;R26R-tdTomato* *Emx1-Cre; pCIG2-Cre; NexCre* *Emx1-Cre; tetO-Cre*	Bcl11b attenuation promotes tumor development through Wnt/β-catenin pathway in ISCs (Sakamaki et al. 2015) and is required for hippocampal development and adult neurogenesis (Simon et al. 2012, 2016).
BAF155	*hGFAP-Cre; Olig2-Cre* *hGFAP-Cre*	BAF155 is required for the specification and proliferation of oligodendrocyte precursors (Abbas et al. 2021) and NSCs proliferation (Nguyen et al. 2018).
BAF170	*hGFAP-Cre; Olig2-Cre* *hGFAP-Cre* *Emx1-Cre; hGFAP-Cre; Nestin-CreER*	BAF170 is required for the specification and proliferation of oligodendrocyte precursors (Abbas et al. 2021) and NSCs proliferation (Nguyen et al. 2018) and differentiation into astrocytes (Tuoc et al. 2017).
BCL7A	*Nestin-Cre; Baf53b-Cre*	BCL7A is required for the differentiation of neural progenitor cells (Wischhof et al. 2022).

**Table 2:** Roles of ISWI, CHD, and INO80 subfamilies in tissue stem cells

Subunit	Cre driver lines	Phenotype in development, regeneration and diseases
**ISWI subfamily**		
SMARCA5	*Vav1-iCre* *heterozygous mutants*	Smarca5 is required in definitive hematopoiesis and self-renewal and differentiation of HSCs (Ding et al. 2021; Kokavec et al. 2017).
BPTF	*Mx1-Cre*	BPTF is required for the maintenance of HSCs (Xu et al. 2018).
**CHD subfamily**		
CHD1	*Vav-Cre*	Specific role for CHD1 during EMT (Koh et al. 2015).
CHD4	*Mx1-Cre* *K14-Cre*	CHD4 is required for the maintenance of HSC quiescence (Yoshida et al. 2008) and early embryonic development of epidermis (Kashiwagi et al. 2007).
CHD7	*Vav1-Cre* *Glast-CreERT2; Nestin-Cre; Rosa-EYFP* *Tlx-CreERT2; Nestin-CreERT2* *Nestin-Cre* *PDGFRα-CreERT*	CHD7 is required in definitive hematopoiesis (Hsu et al. 2020), the maintenance and differentiation of NSCs in adult neurogenesis (Feng et al. 2013; Jones et al. 2015; Micucci et al. 2014) and the differentiation of OPCs (Marie et al. 2018).
CHD8	*Mx1-Cre* *Mx1-Cre* *Emx1-cre; Nestin-CreERT2*	CHD8 is required for stemness and differentiation of HSCs (Nita et al. 2021; Tu et al. 2021) and embryonic and adult neurogenesis (Dong et al. 2022).
**INO80 subfamily**		
ZNHIT1	*Mx1-Cre* *Vav-Cre* *Villin-cre; Olfm4-IRES-eGFPCreERT2*	Znhit1 is required for HSC quiescence (Sun et al. 2020), lymphoid lineage commitment (Ye et al. 2017) and maintenance of Lgr5^+^ ISCs (Zhao et al. 2019).
SRCAP	*Lgr5-Cre; Lgr5GFP-CreERT2*	SRCAP is required for the capacity of self-renewal and epithelial regeneration of Lgr5^+^ ISCs (Ye et al. 2020).
TIP60	*Mx1-cre; Rosa26 CreERT2*	Tip60 is required for HSCs maintenance (Numata et al. 2020).
P400	*Mx1-Cre*	P400 is required for HSC proliferation in adults (Fujii et al. 2010).

### Hematopoietic stem cells

As the most extensively studied tissue-specific stem cells, HSCs residing in the bone marrow are responsible for blood supply throughout the lifetime. The classic model of hematopoietic hierarchy depicts a stepwise process of lineage commitment, and the long-term HSCs (LT-HSCs) at the pinnacle of the hierarchy constitute a dormant population to give rise to short-term HSCs (ST-HSCs) with a restricted capacity for self-renewal within a short period. Subsequently, ST-HSCs can generate multipotent progenitors, MPPs, which further give rise to all lymphoid and myeloid lineages after active proliferation and differentiation (Jacobsen and Nerlov 2019; Wilson et al. 2008).

For all four subfamilies among chromatin remodelers, each one has been firmly implicated in definite roles in the regulation of HSCs’ cell fate decisions up to now. Mammalian SWI/SNF complexes encompass canonical BAF (cBAF), polybromo-associated BAF (pBAF), and non-canonical BAF (ncBAF), all of which require either Brm (SMARCA2) or Brg1 (SMARCA4) as the ATPase motor (Ho and Crabtree 2010). After going through a process known as endothelial-to-hematopoietic transition (EHT), blood progenitors give rise to HSCs during the early embryonic development stage (Patel et al. 2022). Utilizing a Brg1 knockout zebrafish model, Tu et al. vividly demonstrated the specific role of Brg1 in the regulation of definitive hematopoiesis rather than primitive hematopoiesis. The binding of Brg1 to the promoter of KLF2 to activate the *klf2a*-NO signaling pathway provides a mechanism for the reduction in the number of hemogenic endothelial cells, thereby reducing lymphoid and myeloid lineages (Tu et al. 2020). In recent research, it was found that constitutive loss of Brm increased the number of HSCs. However, loss of their self-renewal capacity in transplantation led to HSC exhaustion. Although the role of Brm on HSC expansion is due to cell-extrinsic effects, it can modulate intracellular valine levels to regulate the functions of both HSCs and HPCs (Naidu et al. 2022). The proper functioning of chromatin remodelers underlies their specific role in the cell fate of HSCs. For example, the transcripts of BAF53a (ARP) are enriched in the HSC population compared with more mature lineages in adult BM cells of the mouse hemopoietic system. Deletion of BAF53a in adult mice led to death caused by impaired CDK-dependent self-renewal of HSCs and enhanced apoptosis specifically in myeloid progenitors (Krasteva et al. 2012). As a signature subunit of pBAF complexes, BAF45a (PHF10) deficiency in the adult hemopoietic system also led to a decreased frequency of myeloid progenitors, but the reduction in the number of LT-HSCs cannot be explained by impaired proliferation (Krasteva et al. 2017). Loss of BAF180 in adult mice led to the depletion of the HSC pool, partly by p21-mediated premature senescence, which resulted in the impaired hematopoietic reconstitution of BAF180 KO secondary transplants (Lee et al. 2016). Although loss of ARID2 (known as BAF200, a unique subunit of pBAF) did not reduce the frequency in both hematopoietic stem and progenitor cells (HSPCs), its deletion led to the diminished ability of HSPCs to differentiate into lymphoid lineage upon transplantation without survival blockade, possibly due to the activation of inflammatory pathways (Bluemn et al. 2021). The critical role of ARID2 in lineage reconstitution during competitive transplantation has also been reported in a prior study (Liu et al. 2018). ARID1A (BAF250a) deficiency resulted in the blockade of HSC quiescence by increasing the self-renewal of the HSC population, and the ability to reconstitute and differentiate towards multilineage is severely impaired. Also, the genes affected after ARID1A depletion are demonstrated by ATAC-seq, such as *GATA2*, *CD34*, *CEBPA*, and *CSF1*, being the key regulators of hematopoiesis (Han et al. 2019). As a paralog to ARID1A, conditional loss of ARID1B (BAF250b) in adult mice resulted in impaired myeloid cell reconstitution in competitive transplantation experiments and did not lead to the elevated frequency of HSCs, suggesting a lesser extent of defect compared to ARID1A deficiency (Madan et al. 2023). As an essential subunit in ncBAF, the bromodomain-containing Brd9 has important roles in both normal and malignant hematopoiesis, where Brd9 depletion impaired stemness of HSCs and B cell lineage development, as well as MLL AF9-induced AML initiation and progression. The authors further uncovered the key role of Brd9 in CTCF-mediated chromatin accessibility to promote myeloid differentiation skewing by integrating multi-omics data (Xiao et al. 2023).

Znhit1, also known as p18^hamlet^, and a subunit of the SRCAP complex, is reported to be involved in maintaining the long-term self-renewal capacity of HSCs, thereby preserving their function for multilineage reconstitution. Deletion of Znhit1 in HSCs abrogates HSC quiescence by activating the Pten-PI3K-Akt pathway, consequently resulting in functional exhaustion (Sun et al. 2020). Furthermore, Znhit1 plays a role by interacting with Pcid2 in multipotent progenitors (MPPs) to guide lymphoid lineage commitment. In *Pcid*^−/−^ MPPs, H2A.Z deposition can be achieved at the promoters of lymphoid fate genes by SRCAP, leading to the expansion of lymphoid lineages. Through differentiation in vitro and analysis of *Znhit1*^−/−^ HSCs from reconstituted recipient mice in vivo, the authors showed us an opposite phenotype mediated by Pcid2 deficiency (Ye et al. 2017). Tip60, being a lysine acetyltransferase of P400/Tip60 in mammals, its deletion in both the embryonic and adult stages led to the failure of cell-intrinsic regulation of HSCs via the acetyltransferase activity of Tip60 to regulate hematopoietic reconstitution and genome integrity of HSCs. The global reduction of H2A.Z acetylation and the downregulation of myc targets imply a Tip60-acH2A.Z epigenetic axis for regulating HSC maintenance (Numata et al. 2020). In addition, deletion of p400/mDomino in the adult mouse BM resulted in a decrease in both HSCs and committed lineage cells, which is presumably due to the impaired proliferation of HSCs and apoptotic cell death (Fujii et al. 2010).

Heterozygous mutation or conditional knockout at the onset of definitive hematopoiesis of Smarca5 can specifically impact the development of fetal HSPCs in acquiring the ability to definitive hematopoiesis and the subsequent maintenance of HSPC expansion and differentiation, elucidating the Smarca5-mediated epigenetic programming in the regulation of nascent HSCs to fetal HSCs transition and the expression of critical genes in HSCs (Ding et al. 2021; Kokavec et al. 2017). Recent work presented a new model to precisely study the effect of Smarca5 graded expression by employing a model with a hypomorphic expression of Smarca5, which demonstrates the ability of *S5tg* expression to rescue the defects in early hematopoiesis, the direct effect on HSCs in a competitive transplantation experiment, and the contribution of *S5tg* to the formation of the core ISWI in a tissue-specific manner (Turkova et al. 2024). BPTF, as a subunit in the ISWI subfamily, plays an important role in the maintenance of HSPCs. Conditional deletion of BPTF caused marrow failure, anemia, and leukopenia by downregulating the master “stemness” genes (*Meis1*,* Pbx1*,* Mn1*,* and Lmo2*) along with reduced chromatin accessibility (Xu et al. 2018).

A variety of CHD proteins (CHD1-9) have been discovered to be linked with early embryonic development and the regulation of HSCs. CHD1 exerts its particular effects during the EHT. Deletion of CHD1 in the endothelium led to embryonic lethality at E15.5. Nevertheless, after the EHT, deletion of CHD1 seemed to be dispensable for subsequent development once the HSPC specification was established (Koh et al. 2015). Regarding the NuRD complex (CHD3, CHD4, and CHD5), it is reported that CHD4 regulates the self-renewal capacity and differentiation into the erythroid lineage in a cell-intrinsic manner by modulating the expression of HSC-specific and lineage-primed genes (Yoshida et al. 2008). For the CHD6-CHD9 subfamily, the role of CHD7 primarily in definitive hematopoiesis, but not at the adult stage, to negatively regulate hematopoietic lineage differentiation has been determined. Mechanistically, CHD7 collaborates with RUNX1, a master regulator during EHT, both physically and genetically, to restrain its activity (Hsu et al. 2020). Studies have illuminated the specific role of CHD8 in the regulation of stemness maintenance in HSPCs. Furthermore, loss of P53 significantly rescues the differentiation defects of HSCs to ensure normal hematopoiesis (Nita et al. 2021; Tu et al. 2021).

Here, by distinguishing the functional role of all four subfamilies to regulate HSCs under proliferation or differentiation processes, a preferable role of these remodelers tends to occur in a context-dependent manner in the regulation of fate determination in HSCs.

### Intestinal epithelial stem cells

Lgr5^+^ CBCs at the bottom of the crypts divide to produce progeny cells for gut homeostasis in the adult mammalian gut epithelium (Barker et al. 2007). To validate the stemness of these Lgr5^+^ populations, a mouse model (*Lgr5-EGFP-ires-*CreERT2/R26R-lacZ) was introduced for in vivo lineage tracing (Barker et al. 2007). The lacZ^+^ CBCs stochastically randomly at the crypt base progressively gave rise to lacZ^+^ progeny of all intestinal lineages within 60 days, deeming Lgr5^+^ CBCs as the truly intestinal stem cells. Binding by the R-spondins, Lgr5, and its homologs act as critical receptors in potentiating Wnt/β-catenin signaling to regulate the functions of CBCs during normal development (Carmon et al. 2011; de Lau et al. 2011). Moreover, the capacity of a single Lgr5^+^ CBC to form intact intestinal crypt-villus units further reconfirmed the stemness of Lgr5^+^ CBCs (Sato et al. 2009).

Deficiency of Brg1 in small intestinal epithelium resulted in the depletion of the stem cell population, followed by compromised proliferative capacity in small intestinal stem cells, ultimately resulting in crypt ablation. It should be noted that loss of Brg1 has a negligible impact on transit-amplifying (TA) and other differentiated cells. In contrast to Brg1-mediated self-renewal in small intestinal stem cells for the establishment of small intestinal homeostasis, the preservation of Brg1-deficient crypts in large intestinal epithelium for an extended period has shed light on the distinct role of Brg1 in a context-dependent manner (Holik et al. 2013). A subsequent study revealed the effects of Brg1 deletion in the murine intestinal epithelium, which led to early post-natal death with impaired morphogenesis and differentiation, could be rescued by Notch1 ICD overexpression, and further indicated the involvement of other signaling pathways due to the inability to rescue stem cell loss in duodenum (Takada et al. 2016). Hiramatsu et al. demonstrated the essential role of one subunit of the SWI/SNF complex, Arid1a, in the regulation of self-renewal of Lgr5^+^ ISCs in the small intestine. Loss of Arid1a in intestinal epithelial cells also causes increased apoptosis and skewed differentiation. CHIP-seq and functional assays indicate that Arid1a directly regulates Sox9 expression, which is further verified by the restored phenotypes through Sox9 overexpression in *Villin-Cre; Arid1a*^f/f^ mice (Hiramatsu et al. 2019). Identified as a core member in the SWI/SNF complex and a haploinsufficient (intolerant to the loss of a single allele) tumor suppressor, Bcl11b attenuation in Apc^min/+^ mice promotes intestinal tumorigenesis, which may be attributed to the increased expression of Wnt/β-catenin targets in Bcl11b-enriched Lgr5^+^ CBCs. Moreover, allelic loss and mutations in human colon cancers also strongly suggest the critical contribution of Bcl11b to human colorectal tumorigenesis (Sakamaki et al. 2015).

The INO80 subfamily also executes its function in intestinal stem cells. Chromatin remodeler Znhit1, which is a subunit of the SRCAP complex, was first reported to participate in maintaining mammalian intestinal homeostasis and cell fate determination in 2019. Mechanistically, the Znhit1-mediated incorporation of histone variant H2A.Z at the TSS regions of Lgr5, Tgfb1, and Tgfbr2 regulates Lgr5^+^ ISCs self-renewal by supporting YL1 phosphorylation (Zhao et al. 2019). The phenomenon that deletion of Znhit1 disrupted the postnatal generation of Lgr5^+^ ISCs indicates an important role of SRCAP-mediated intestinal crypt establishment after the completion of embryonic development. Interestingly, deletion of the catalytic subunit, SRCAP, led to impairment of self-renewal maintenance and epithelial regeneration capacity upon irradiation injury of Lgr5^+^ ISCs and demonstrated that the active SRCAP complex recruits REST to initiate the transcription of Prdm16, thereby activating PPARδ for the maintenance of ISC stemness (Ye et al. 2020).

In the Drosophila intestine, the BAP SWI/SNF complex, defined by OSA (ARID1), assumes critical roles in the balance of ISCs between self-renewal and their differentiation into EE cells and EC cells. Specific deletion of OSA in ISCs and EBs resulted in the expansion of ISCs, but not EBs, in the posterior midgut. Mechanistically, the OSA in ISCs promotes the differentiation of EB to EC through the Dl-N signal axis, while the OSA governs EE differentiation by regulating the EE cell fate regulator ASE, rather than the previously proposed notion that the N signal is required for EE cell fate determination (Zeng et al. 2013). The core component of the SWI/SNF complex, Brm, also plays an important role in cell fate determination of ISCs, and deletion of Brm resulted in differentiation defects of ISCs into ECs. More importantly, Brm serves as a downstream target of the Yki-sd axis to maintain the proliferation ability of ISCs during DSS-induced intestinal regeneration, a process in which Hippo signaling exhibits direct regulatory functions (Jin et al. 2013).

In summary, chromatin remodelers have been shown to be involved with chromatin control of key genes during cell fate determination in IESCs by collaborating with transcription factors, histone variations, and signaling pathways.

### Neural stem cells

In the mammalian brain (central nervous system), neurogenesis occurs at the subventricular zone (SVZ) of lateral ventricles and subgranular zone (SGZ) of the dentate gyrus (DG) in the hippocampus, which is regulated by neural stem cells (NSCs) and neural progenitor cells that finally differentiate into varied neural cells, including neurons and glia (astrocytes and oligodendrocytes) (Bond et al. 2015).

The BAF complex is a notable regulator of neural development and has been implicated in performing important roles in NSCs. During the transition from neurogenesis to gliogenesis, epigenetic changes in cell fate choices for NSCs at this stage are of great importance. Disruption of BAF155 and BAF170 expression in a double knockout mouse model led to impaired proliferation and specification of oligodendrocyte precursors, which finally affected the production of oligodendrocytes in the mouse forebrain (Abbas et al. 2021). The role of double-conditional knockout of BAF155 and BAF170 in the regulation of the balance between self-renewal and proliferation in NSCs has also been implicated in a prior study (Nguyen et al. 2018). Conditional knockout of BAF170 in adult neurogenesis broke the maintenance of NSCs to differentiate into astrocytes rather than neuronal progenitors (Tuoc et al. 2017). As a subunit in the SWI/SNF complex, Bcl11b is required for both embryonic and adult neurogenesis (Simon et al. 2012, 2016). It is worth mentioning that Brg1 is specifically expressed in cortical SVZ between E14 and birth, and loss of Brg1 did not affect early neuronal differentiation in the mouse brain. However, once committed to neuronal lineages, Brg1 deficiency in these NSCs could result in consequent differentiation failure. Specifically, Brg1 loss of function blocks the differentiation of NSCs into astrocytes and some oligodendrocytes, rather than the maintenance of survival after the establishment of astrocyte differentiation (Matsumoto et al. 2006). The critical role of Brg1 has also been investigated during the early stage of neurogenesis rather than the postnatal stage of NSC maintenance (Jin et al. 2022), and the process by which Brg1 acts to regulate the stemness of NSC is large via a p53/p21-dependent process (Petrik et al. 2015). What’s more, Brg1 directly interacts with the Olig2 promoter and represses its expression in neurogenic progenitor cells, and loss of Brg1 in NPCs leads to ectopic Olig2 expression in the cortex, and diminished ability to differentiate into oligodendrocyte lineage cells, which indicates a distinct role for Brg1 in the specification and differentiation of OPCs, respectively (Matsumoto et al. 2016). This work well extends the role of SWI/SNF complex in oligodendrocyte differentiation and maturation described in a previous study, where Brg1 is recruited by the determination factor Olig2 to cis-regulatory elements of essential factors responsible for oligodendrocyte differentiation, such as Sox10 (Yu et al. 2013). Contrary to the above conclusion that Brg1 is required for oligodendrocyte differentiation, one study suggested that Brg1 is dispensable for the differentiation and maturation of oligodendrocytes. Nevertheless, Brg1 did have a role in regulating the number of oligodendrocytes at an early stage. The different conclusions may be due to the cre driver lines the authors used in their studies (Bischof et al. 2015). Deletion of Brg1 caused the differentiation conversion of adult NSCs from neuronal progenitors to glia, highlighting an important role for the PAX6-BAF complex in the initiation of a regulatory network essential for the maintenance of the neurogenic lineage in the adult brain (Ninkovic et al. 2013). Regarding developmental disorders, the BAF complex is implicated in the prevention of miR-9 loss in NSCs following ethanol exposure, which provides important insights into further access to the role of the BAF complex in prenatal alcohol exposure (Burrowes et al. 2017). Except for the core part of the BAF complex, Brg1, other compartments in Brg1-containing BAF complex, like BAF250A (ARID1A), also perform an important role in neurogenesis, during which conditional knockout of ARID1A in forebrain neural stem/progenitor cells led to the inhibition of proliferation in radial glial progenitors and their differentiation (Liu et al. 2021). Notably, the disruption of ARID1A perturbed the release of PRG3 in microglia (resident macrophages in the central nervous system), leading to dysregulation of the self-renewal and differentiation of neural progenitors, which finally led to autism-like behaviors at later stages (Su et al. 2024). A component of the BAF complex, BCL7A, plays critical roles in regulating neurogenesis by potentiating Wnt signaling and mitochondrial bioenergetics in neural progenitor cells, and in supporting animal behavioral performance (Wischhof et al. 2022). By performing whole-exome sequencing in a large patient cohort of a total of 2697 patients as well as using a Xenopus tropicalis model of congenital hydrocephalus, Singh et al. suggested that SMARCC1 is important for human congenital hydrocephalus pathogenesis, supported by a “neural stem cell” paradigm (Furey et al. 2018; Singh et al. 2024). What is worth mentioning is that the subunit exchange of chromatin remodeler during neurogenesis is of great significance in neural development (Lessard et al. 2007).

CHD7, with its high expression level in the adult mouse brain, conditional loss of Chd7 in active NSCs in both the SVZ and SGZ regions led to defects in neurogenesis. Furthermore, CHD7 keeps the promoters of essential regulators, Sox9 and Sox11, in an open chromatin structure, and overexpression of either of these transcription factors could largely rescue the aberrant differentiation (Feng et al. 2013). Consistent with CHD7 being required for adult neurogenesis, CHD7 is essential for the proliferation and differentiation of NSCs in the SVZ during both embryonic and adult neurogenesis by directly regulating the RA receptor. Modulation of RA signaling can attenuate the defects in the absence of Chd7, which suggests possible curative therapies for CHARGE-related defects (Micucci et al. 2014). What’s more, CHD7 also participated in the maintenance of NSC quiescence to prevent the conversion of these NSCs into immature lineage-restricted progenitors. Mechanistically, Chd7 regulated the notch gene Hes5 to induce NSC quiescence, and loss of Chd7 led to the downregulation of Hes5 and the subsequent depletion of the NSC pool (Jones et al. 2015). In adult brains, CHD8, the high-confidence autism gene, is required for neurogenesis via intermediate progenitor cells (IPCs) in both forebrain SVZ and hippocampal SGZ rather than the survival of NSCs (Dong et al. 2022). Additionally, the dynamic regulation of CHD7/CHD8, in cooperation with Olig2/Sox10, in the regulation of oligodendrocyte precursor cells (OPCs) proliferation, survival, differentiation, lineage progression, and maturation during neurogenesis shed light on the very specific temporary-dependent role of chromatin remodelers in cell fate determination (Marie et al. 2018).

Taken together, both the SWI/SNF and CHD subfamilies have been implicated to function in NSCs during development and throughout adulthood to control the precise developmental timing and cell fate determination, whereas it could lead to disorders when these factors are disrupted.

### Skin stem cells

Our skin system consists of the epidermis, dermis, hair follicle, and sebaceous gland. Stem cells that reside in the basal layer of the interfollicular epidermis (IFE) and the hair follicle bulge in the adult skin are responsible for the rapid turnover of the mammalian epidermis. During hair formation in each regenerative cycle, dynamics regulation of growth, regression, and quiescence is controlled by hair follicle stem cells (Blanpain and Fuchs 2006). It is noted that the label-retaining hair follicular epithelial stem cells in the bulge could generate all bugle structure and also IFE whenever after damage or being transplanted (Ito et al. 2005); nevertheless, under homeostasis, they could only generate the bulge part, which makes the keratinocyte a more functional cell type under different conditions, with the emphasis on its ability as a true quiescent stem cell whenever needed to be activated upon injury.

ACTL6a, also known as BAF53a; deletion of ACTL6a in adult epidermis led to loss of maintenance of epidermal progenitors and derepressed the activation of differentiation genes, such as KLF4. Under homeostasis, ACTL6a maintains the progenitor state by abrogating the function of SWI/SNF to bind to the promoters of differentiation genes (Bao et al. 2013). Given the important role of hair follicle stem cells in giving rise to all epidermal compartments, studies have focused on the mechanisms for hair regeneration. Another study showed that Brg1 is required for hair regeneration under homeostasis and during repair. Postnatal deletion of Brg1 caused impaired hair regeneration during growth due to the lack of a stem cell pool. Mechanistically, this feedback loop between Brg1 and Shh, in which dynamics regulation of Brg1 expression is partly determined by Shh signals through Gli and activation of Shh expression is dependent on the cooperation of Brg1 and NF-kB at the Shh promotor (Xiong et al. 2013). As a direct transcriptional target gene of the lineage-specific transcription factor p63, Brg1 makes a substantial contribution to the differentiation of epithelial progenitor cells during development by regulating the relocation of the epidermal differentiation complex EDC towards the nuclear interior and gene expression within the EDC locus, cooperating with other important transcription factors (Mardaryev et al. 2014). Also, the depletion of Mi-2β (CHD4) at different early embryonic stages reveals a requirement for Mi-2β at three critical transitions: the self-renewal capacity of epidermal precursors in the basal epidermis, the fate conversion of the basal epidermal cell to the follicular cells, and the subsequent differentiation of follicular progenitors to matrix stem cells (Kashiwagi et al. 2007).

### Mesenchymal stem cells

Mesenchymal stem cells of adult or embryonic sources are conferred a distinct ability to differentiate into multiple lineages, mesodermal lineage includes adipocytes, osteocytes, and chondrocytes from adults, in which the bone marrow is the main type of MSCs with a great ability to differentiate towards the mesodermal lineage (Méndez-Ferrer et al. 2010; Muguruma et al. 2006).

For the induction of osteocyte and adipocyte lineages, the SWI/SNF subfamily is responsible for their differentiation. Studies have shown the potential of chromatin remodelers to improve the differentiation efficiency of MSC. Brg1 participates in forming transcriptionally active complexes together with p300, which can be stabled by phosphorylation of Osx by p38 at Ser-73/77 (Ortuño et al. 2010). The role of the pBAF complex and pbrm1 in MSC osteogenic differentiation reveals their importance in integrating BMP/TGF-b signaling and chromatin remodeling to regulate osteogenesis and hematopoiesis (Sinha et al. 2020). Overexpression of Brg1 contributes to the differentiation of MSC into adipocytes (Napolitano et al. 2007). Besides, the CHD subfamily and ISWI subfamily have also been implicated in the osteogenesis of MSC. CHD1 has been reported to regulate transcriptional changes during osteogenic differentiation of MSC. Depletion of CHD1 resulted in suppression of differentiation-activated genes in osteoblasts as well as adipocytes due to increased pausing of RNA Polymerase II (RNAPII) and decreased H2A.Z occupancy close to the TSS (Baumgart et al. 2017). For CHD7, its role in osteogenic differentiation of MSC to interact with SMAD1 could increase osteogenic ability (Chen et al. 2016). The osteogenic capability of MSC is compromised by silencing INO80 both in vitro and in vivo (Zhou et al. 2016).

In a word, by modifying ATP-dependent chromatin remodeling complexes, people enhance the efficiency of differentiating MSCs into a variety of cell types. Still, more research is needed.

## Conclusions

A key characteristic of TSCs is their significant role in maintaining tissue homeostasis. Their importance in restoring tissue integrity has made them the most potent source in regeneration medicine. Compared to ESCs, TSCs show greater safety and ease of manipulation and can more accurately differentiate into a specific organ or tissue without immunological rejection. However, the regulatory mechanisms governing these processes remain nebulous. What constitutes their fundamental ability to restore tissue loss, and what are the similarities among distinct subpopulations of stem cells? Informative messages can be readily disregarded without the precise simulation of tissue injury and the corresponding cellular behaviors as manifested in biological contexts. The exploration of the characterization of TSCs by the next generation is extremely important to comprehensively identify the specific regulators modulating their behaviors at a given developmental time.

Epigenetic regulation of cell fate decisions plays a critical role for TSCs during development, the maintenance of homeostasis, and the protection of individuals against diseases. These regulatory mechanisms constitute important modulators that guide cellular behavior, ensuring proper tissue function and repair. The promotive role of chromatin organization regulators in regulating other contributors, such as DNA methylation (Jeddeloh et al. 1999), possesses considerable importance in eukaryotic gene regulation. Studies have revealed the activities of DNA methylation and other histone modifications in TSCs (Avgustinova and Benitah 2016), and additional research is warranted to fulfill the molecular basis of chromatin remodeling by chromatin remodelers in the regulation of TSCs. Tools developed in recent years, such as Hi-C, have helped us discover more and more possible participating factors in chromatin factors-mediated regulation of cell fate determination of TSCs (Takayama et al. 2021). These technologies will undoubtedly assist in deciphering the “epigenetic code” in TSCs. Chromatin remodelers play a crucial role in development, diseases and regeneration, and the basic mechanism underlying the regulation by four subfamilies is their ability to change chromatin states, thus enabling the transcription machinery to be accessible to chromatin. Different subfamilies exert their specific effects in regulating tissue stem cell fate determination in developmental stages by modulating distinct regulators in a given tissue. In the regulation of TSCs, ATP-dependent chromatin remodeling can modulate the fate of these cells through dosage-dependent (Krasteva et al. 2017) and haploinsufficiency (Sakamaki et al. 2015) mechanisms, consistent with their role in the development of disease (Morrill and Amon 2019; Rice and McLysaght 2017). This suggests the need to investigate the dosage sensitivity of critical subunits in the context of TSCs to understand their specific roles in disease and to develop precise targets for therapeutic intervention. Notably, the fact that these complexes are combinatorially assembled from different subunits into one subfamily or different subfamilies has endowed them with multitasking potential in the regulation of appropriate control of gene expression to enable the cell fate determination of specific lineages. Besides, the four families of chromatin remodeling complexes are typically genetically non-redundant in mammals. Loss-of-function mutations in one gene could lead to adverse effects, particularly during early embryonic development (Marfella et al. 2006; Bernier et al. 2014). From the perspective of TSCs, loss of function of these critical subunits indeed impacts the function of multiple types of stem cells and ultimately leads to diseases, which helps us identify important subunits that affect TSCs to better establish a foundation for further research. On another level, these chromatin remodelers mediate different biological processes involved in the regulation of stem cell functions, either directly or indirectly influencing the subsequent gene transcription, which requires the participation of a large number of other factors. It is important to note that the intricate crosstalk between chromatin remodelers and other factors is perceived as relatively rudimentary, far from comprehensively figured out, and remains to be investigated. Being implicated in many disorders, for example, a recent study indicated that a competitive advantage in SRCAP mutant HSCs enriched in patients following genotoxic stress led to the development of clonal hematopoiesis with a lymphoid-biased expansion (Chen et al. 2023), the precise modulation by chromatin remodelers is a critical determinant in the fate control of TSCs, which can tell us that the dysfunction of chromatin remodelers could lead to the disruption of tissue homeostasis, predisposing individuals to disorders, developmental diseases and cancer. Future genetic studies will elucidate how the chromatin remodelers coupled with other chromatin remodeling proteins work together in tissue stem cell fate determination, which can provide potential strategies for diseases driven by chromatin remodeling defects, more thought should be given to how to develop more personalized and effective treatment strategies based on an in-depth understanding of chromatin remodeling and regulatory mechanisms, such as using new imaging technologies (Lucignani et al. 2006; Ertl et al. 2014) to monitor the activities and changes of TSCs more accurately or using gene editing techniques (Maeder et al. 2016) to correct abnormal genes in TSCs more directly, thereby providing a real-time basis for the formulation of treatment plans. TSCs reside in specific organ such as bone marrow-derived MSCs have shown therapeutic effects with their broad properties such as multilineage differentiation and easy isolation, which makes them a suitable source for cell therapy to improve diseases and regeneration (Margiana et al. 2022). A comprehensive understanding of chromatin remodelers that govern tissue stem cell fate determination, along with the consequences of their disruptions by using animal models, is imperative for developing effective therapeutic interventions to target chromatin remodelers, particularly the SWI/SNF complex, that are mutated in human cancers. Numerous drugs aimed at these mutations have demonstrated encouraging outcomes in preclinical tumor models and clinical advancements include FDA-approved EZH2 inhibitors for cancers with SMARCB1 mutations and more targets are now identified through genome-wide screens for clinical trials (Malone and Roberts 2024). Therefore, the roles of chromatin remodelers in TSCs help us further understand how the enhanced or reduced activity of specific subunit mutation in select cell populations promotes diseases and approaches for targeting the structure and function of chromatin remodelers are developed to enhance therapeutic benefits (Centore et al. 2020).

## Data Availability

Not applicable.

## Abbreviations

MSCs: Mesenchymal stem cells
TSCs: Tissue stem cells
HSCs: Hematopoietic stem cells
MuSCs: Muscle satellite cells
NSCs: Neural stem cells
HFSCs: Hair follicle stem cells
IESCs: Intestinal epithelial stem cells
INO80: Inositol requiring 80
ISWI: Imitation switch
CHD: Chromodomain helicase DNA-binding
SWI/SNF: Switch/sucrose non-fermentable
OPCs: Oligodendrocyte precursor cells
CBCs: Crypt base columnar cells
EHT: endothelial-to-hematopoietic transition
IFE: Interfollicular epidermis
SGZ: Subgranular zone
SVZ: Subventricular zone
